# Chased and displaced: Impact of anthropogenic disturbance on habitat use and movement patterns of Asian elephants in South West Bengal, India

**DOI:** 10.1016/j.isci.2026.117480

**Published:** 2026-09-21

**Authors:** Aakriti Singh, Honnavalli Nagaraj Kumara

**Affiliations:** 1 Sálim Ali Centre for Ornithology and Natural History, Anaikatty Post, Coimbatore 641108, Tamil Nadu, India

**Keywords:** human-elephant conflict, habitat use, elephant movement ecology, conflict mitigation strategies, hula drive, adaptive governance, community-mediated management

## Abstract

Managing large mammals sustaining on crops in human-dominated landscapes is challenging, and the most common mitigation strategy is “driving”. In South Bengal, elephants are driven away by locals or organized “*hula*” teams. Over 223 monitored days in 2017–18 (56 no drive, 97 local, and 70 *hula* drives), we collected data on elephant movement, habitat use, costs, and community responses. Mean monthly movement type differed significantly, with local drive being highest (0.43 km ± 0.10 SD), followed by *hula* (0.31 km ± 0.11 SD) and no drive (0.26 km ± 0.12 SD). Mean daily path length differed too, *hula* drives being highest (9.43 km ± 4.24), while local drives were lowest (5.20 km ± 2.34). Expenditure on *hula* drives correlated positively with *hula* size (5.86 ± 2.00 SD). Drives were mainly triggered by grievances (54%) and crop damage (24%). Local communities interfered in 55.71% of drives. *Hula* drives are reactive, socially mediated, energy-intensive interventions offering only short-term relief, not a long-term mitigative measure.

## Highlights

• *Hula* drives result in longer daily path lengths than local drives and natural movement
• Herd size and hula size are negatively related to daily path length during *hula* drives
• Human attacks peak on *hula*-drive day, while crop depredation rises afterward
• Community response to *hula* drives is mixed, with more interference than assistance

## Graphical abstract

**Figure:**
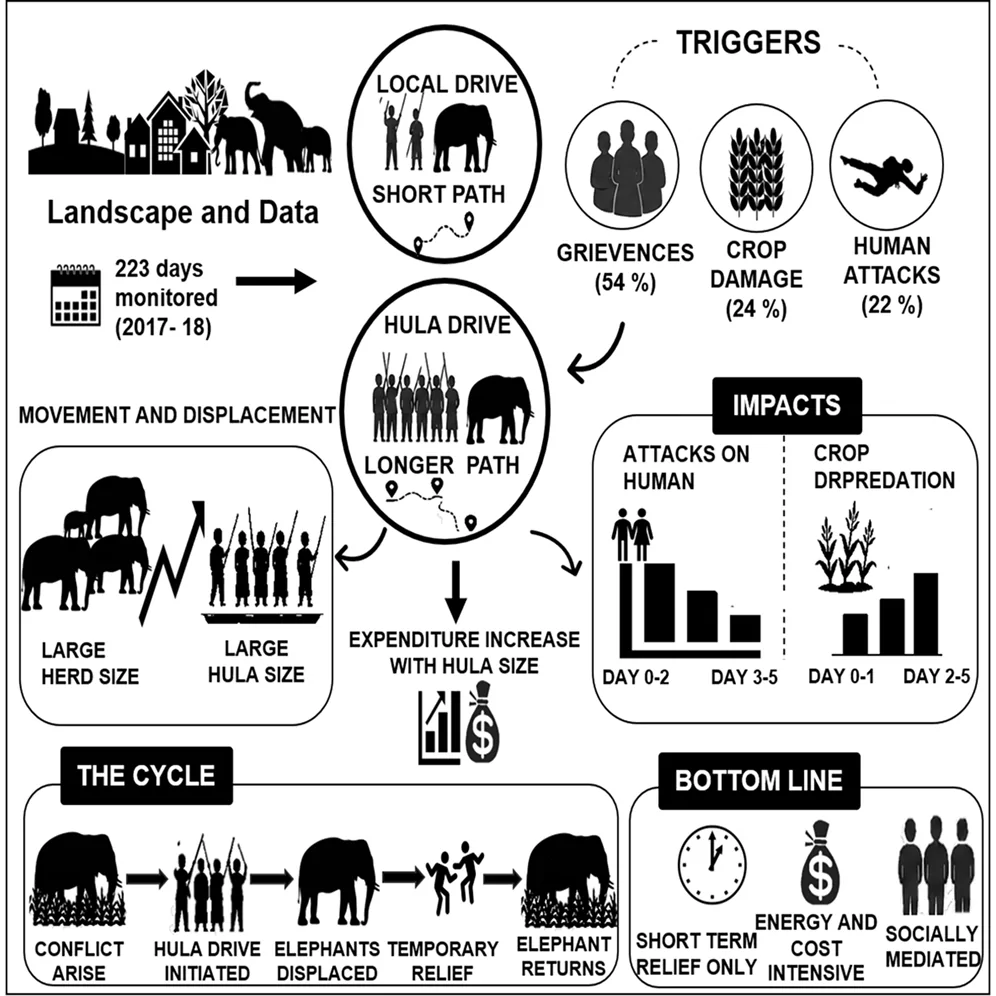

## Teaser

Wildlife behavior; Environmental management; Ecology; Zoology

## Introduction

Animal movement is an essential ecological process that governs species distribution, population dynamics, and ecosystem stability.^1^ This phenomenon is formed by complex interactions of both biotic and abiotic factors acting across multiple spatial and temporal scales.^2^ An understanding of the movement patterns of migratory species is crucial for revealing their spatial distributions, resource utilization, and behavioral adaptations,^3^ while simultaneously providing the ecological knowledge.^4^^,^^5^^,^^6^ Movement dynamics are shaped by various environmental factors, including habitat quality, food availability, predation risk, and anthropogenic disturbances, further influencing the frequency, directions, and extent of movement.^7^

Human activities, such as land-use alterations and infrastructure expansion, constrain animal movements^8^ and increase the likelihood of human wildlife interactions.^9^ The increase in infrastructure, agriculture, and human settlements across the globe increases the probability of negative human-wildlife interactions, especially in areas adjacent to protected areas.^10^ Human-elephant conflict (HEC) has become a conservation issue in areas where economic losses due to crop depredation, property damage, and raising threats to human safety^11^^,^^12^ often prompt retaliatory killings of elephants, which further threaten the elephant population.^13^^,^^14^

Various HEC mitigation strategies have been implemented worldwide, with varying degrees of success and sustainability, including “elephant drive,” which is a widely used mitigation measure and requires actively driving elephants away from human settlements while guiding them toward forests.^15^ This practice is often coupled with the use of deterrents like bright lights, firecrackers, loud noises, or chili-based repellents to keep the elephants away.^16^^,^^17^ The driving of elephants has been practiced in many parts of Asia, e.g., Sri Lanka^18^ and Thailand.^19^ Another way utilizes trained elephants (known as *kunkis*) to guide wild elephants away from conflict areas.^20^ However, the success of these drives is often challenged by several factors, including gradual habituation by elephants to *kunkis* and deterrents, reduced responsiveness to deterrents, and often aggressive approaches toward humans.^15^^,^^18^^,^^21^ In many cases, the driving of elephants often results in losing part or the entirety of their home range, leading to inadaptability to new areas due to loss of familiar resource locations, and refuge sites, and elevated stress causing high morbidity and mortality among the animals,^16^^,^^22^ while increasing conflict manifold. Despite their prevalence, these interventions are rarely evaluated beyond immediate conflict resolution, resulting in limited understanding of their effectiveness, behavioral consequences, and animal welfare implications.^23^

In India, organized elephant drive, usually of two types—local drives and *hula* drives (named after “*hula*” which is a long wooden spear with a sharp metal spike sticking out of the top and a gunny sack attached and set ablaze), involving forest staff and local communities, are primarily used to displace crop-raiding elephants from agricultural fields back into forested areas. The practice is logistically inexpensive, socially rooted, and often perceived as immediately effective, contributing to its continued use across eastern and northeastern India.^24^ However, concerns persist regarding human safety, elephant stress, and the long-term effects of repeated drives, particularly in landscapes characterized by severe conflict.

From a global perspective, organized elephant drive is not unique but constitutes a context-specific variant of non-lethal elephant displacement strategies used worldwide. The concept of *hula* drives is not just specific to India, drives similar to *hula* drives occur across all the elephant landscapes. Comparable approaches include problem animal control (PAC) drives in Sri Lanka,^18^ and coordinated chase-based deterrents in African agricultural landscapes.^25^ These methods share an implicit assumption that short-term spatial displacement reduces conflict without incurring long-term ecological or behavioral costs, an assumption that remains poorly tested empirically.

Emerging research in elephant behavioral ecology highlights the importance of movement energetics, social structure, and stress responses in shaping elephant reactions to anthropogenic disturbance.^26^^,^^27^ Repeated or intensive displacement may impose energetic costs, disrupt herd cohesion, and influence future ranging behavior, potentially increasing the likelihood of recurrent crop raiding.^28^ Yet, such behavioral dimensions are seldom quantified in assessments of crowd-based drives involving repeated human pressure.

In this context, the present study in South West Bengal (Figure 1) evaluates organized elephant drives as a case study of global relevance, focusing on their effects on elephant movement behavior and herd-level responses. By quantifying parameters such as path length (PL), herd size (HS) effects, and post-drive movement patterns, this study addresses a critical gap in the evidence base for non-lethal elephant displacement strategies. The study places the organized elephant drive in an adaptable, empirically supported framework for HEC management rather than evaluating the practice only in terms of instantaneous conflict resolution. The main aim of this study was to evaluate the effectiveness of organized hula drives as a potential conflict mitigation tool through changes in the movement behavior of the elephants, habitat use, and socio-political factors in human-dominated areas. By linking a regionally specific intervention to globally shared challenges in elephant conservation, this study contributes to international debates on the effectiveness, sustainability, and ethical dimensions of reactive conflict mitigation, with implications for elephant-range landscapes beyond India.

**Figure 1:**
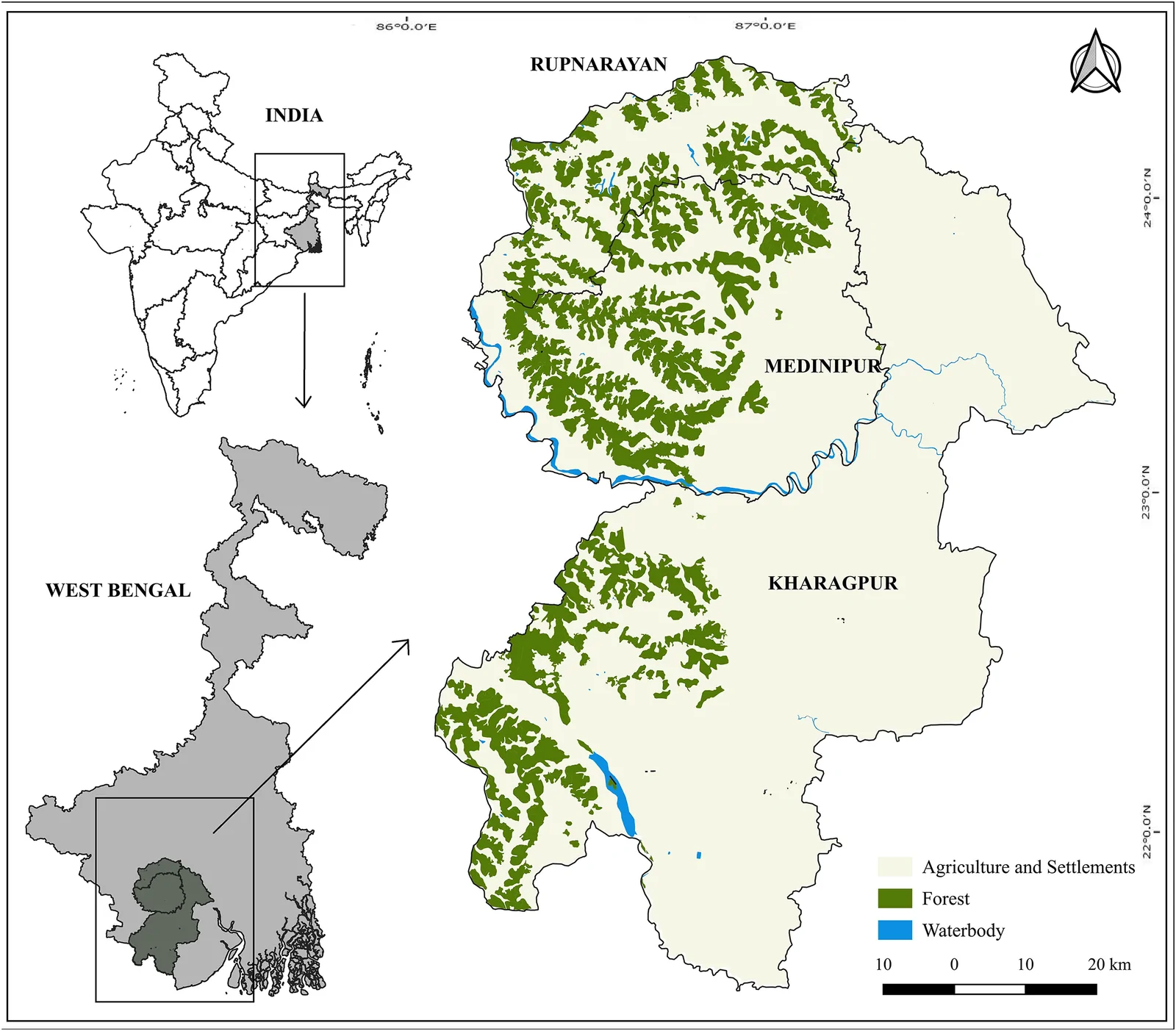
Forest cover in Kharagpur, Medinipur, and Rupnarayan forest divisions of South Bengal, India

## Results

### Elephant movement pattern

The elephant movement without any interference for a complete day was considered natural movement, which is mentioned as “no drive,” while the movement that was decided by the people was considered a drive. We observed two types of elephant drives: the local drive and the organized drive “*hula* drive.”

Local drive: local people drove away elephants whenever elephants were near their crop fields. The drive was conducted by only a few people using firecrackers and loud noises, typically for a few hours (3–4 h) and concluded upon relocation of the elephant from its current location, usually to the nearby village or cropland.

*Hula* drive: the Forest Department specifically deployed a group of trained personnel to relocate herd of elephants from conflict area to distant forest. During the driving, the elephants were scared away using “*hula*” (a long wooden spear with a sharp metal spike sticking out of the top and a gunny sack attached, which is set ablaze), a popular management strategy used in this landscape. Each person involved in the drive was given a *hula*, crackers, and handheld torches. Forest Department personnel coordinated the drive, and followed the *hula* drive by vehicles with the required additional support, including mobile oil (waste engine oil in drums in the vehicle), crackers, and a drum. All the *hula*-party members and officials met at one location, prepared the *hula*, and developed a drive strategy to push and guide the elephants toward the identified forests. They executed the *hula* drive in a synchronized manner using loud noises and *hulas* to encourage the elephants to move in the intended direction. The elephant herd occasionally split into several groups and traveled in different directions. In those cases, either the *hula* party broke up too or reinforcements were sent in from other forest ranges to assist the divided party to join them so that the herds were successfully pursued in one direction. The *hula* usually lasts an entire night (10–11 h). Once the driving was completed, the *hula* party and forest officials left the elephants undisturbed.

Money spent on the wages for the *hula* personnel, mobile oil, food for the *hula* members, crackers, drums, and transportation varies from around 3 lakh/night to 9 lakh/night (*n* = 21, 5.86 ± 2.00_SD_ lakh/night). The money spent on *hula* drive is related to the *hula* size (*r*_s_ = 0.830, *N* = 21, *p* < 0.001), but money spent on *hula* drive was not related to elephant HS (*r*_s_ = 0.007, *N* = 21, *p* = 0.976) or their driving distance (*r*_s_ = −0.270, *N* = 21, *p* = 0.237). Of the 70 *hula* drives witnessed, the grievance by local people remained the major reason for driving away elephants (54%), followed by crop damage (24%), human injuries (13%), and human deaths (9%) (Figure S1).

### Movement pattern and habitat use by the elephant

#### Habitat use

Of the 1,959 grid cells, elephants used 182 (9.29%) grid cells during the 223 days of movement in the study period by herds 1 and 7. 112 grid cells were utilized by elephants during the days of *hula* drives, 126 grid cells during local drive days, and 106 grid cells during no drives (Figure 2). The overlap in the use of the grid cells slightly differed between the movement types: the overlap between all the movement types was 47 grids, while it was 73 between *hula* and local drive, 76 grid cells between local drive and no drive, and 57 between *hula* and no drive. Friedman test for k-related measures showed no significant difference in grid usage by elephants among the three driving types (χ^2^ = 1.47, *N* = 182, and *p* = 0.479).

**Figure 2:**
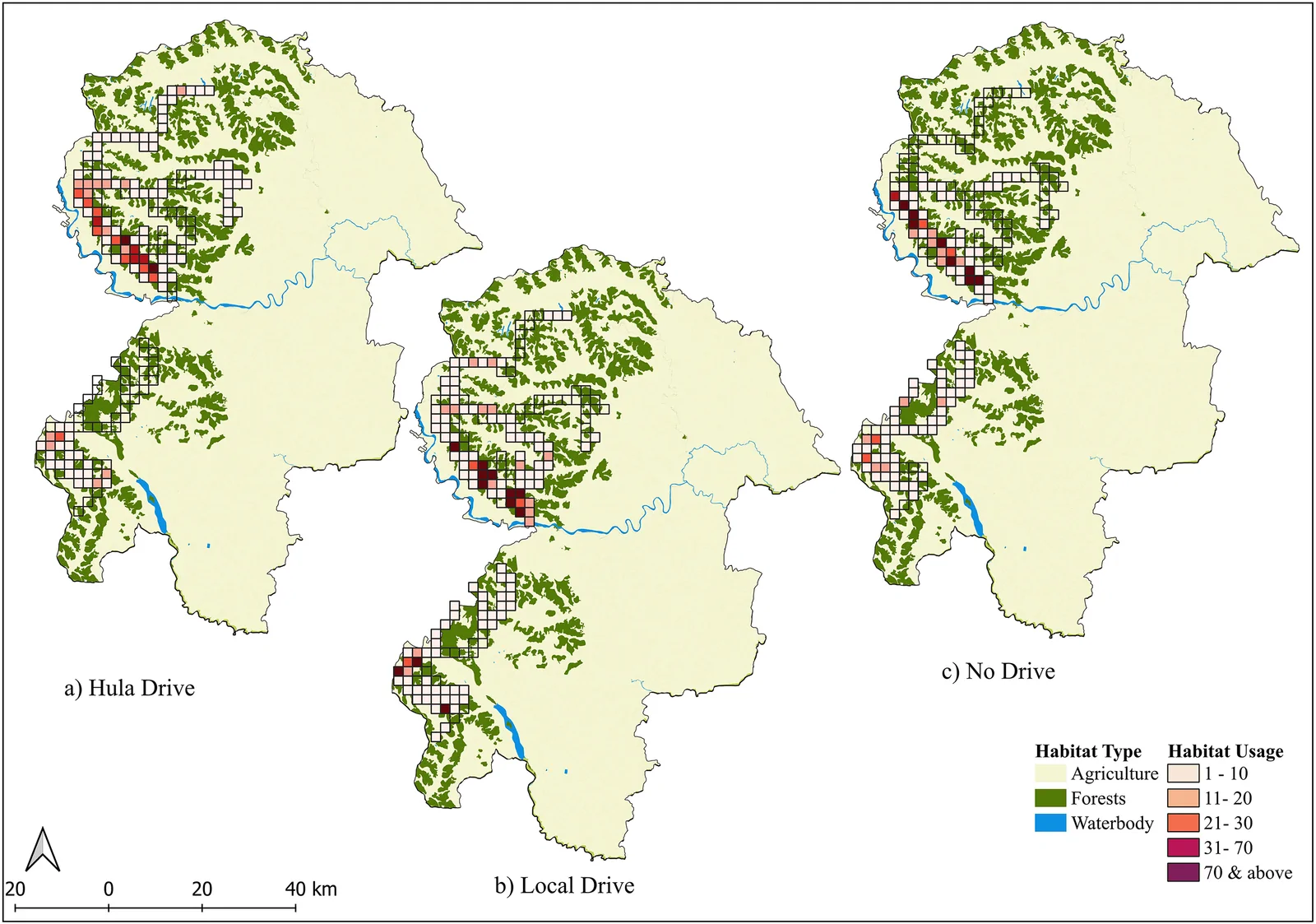
Habitat use by selected herds of elephants in South Bengal during different movement patterns (A) Hula drive, (B) Local drive, and (C) No drive.

#### Movement pattern

We recorded 223 days of movement data, of which 56 days had no drive, 97 days had local drive, and 70 days had *hula* drive (for all the herds). We recorded the *hula* drive and local drive throughout the year; however, the *hula* drive was relatively higher during the months of April to September (pre monsoon and monsoon period) (Figure S2), and the local drive was consistent throughout the year, peaking in March. Mean proportion of days with local drives in a month was 0.43 ± 0.10_SD_, which was followed by *hula* drive (0.31 ± 0.11_SD_) and no drive (0.26 ± 0.12_SD_), which differed significantly (F_10_, _46_ = 7.689, *p* < 0.002).

Mean PL during the *hula* drive (9.43 km ± 4.24_SD_) was higher than during the natural movement (6.81 km ± 5.27_SD_) and local drive (5.20 km ± 2.34_SD_), and differed significantly among movement types (F_2_, _223_ = 24.352, *p* < 0.001) (Figure 3). The mean PL between the months during *hula* drive (F_10_, _59_ = 1.985, *p* = 0.51) and local drive (F_10_, _86_ = 1.557, *p* = 0.13) did not differ significantly; however, it differed during the no drive (F_10_, _45_ = 4.421, *p* < 0.01) (Figure 4). Because multiple daily observations were collected from the same herds, our analysis may overestimate degrees of freedom. During the monitoring period, elephants showed longer daily paths during hula drives than during local drives or no-drive conditions. This pattern is consistent with repeated observations from intensively monitored herds and should be interpreted as a descriptive comparison, not as an inference about the population level.

**Figure 3:**
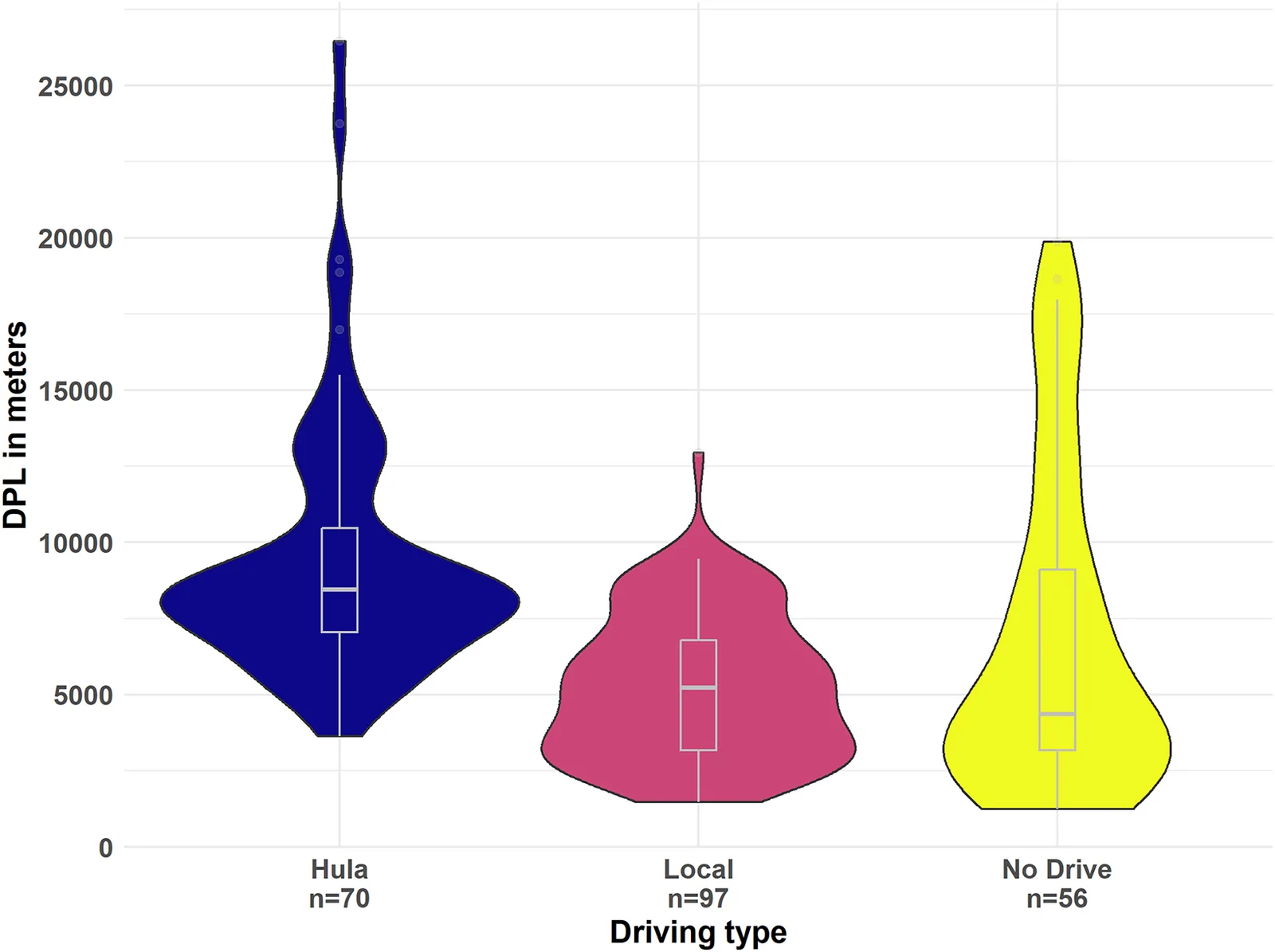
The path length of elephants in different drive types in South Bengal Error bar represent median with SD.

**Figure 4:**
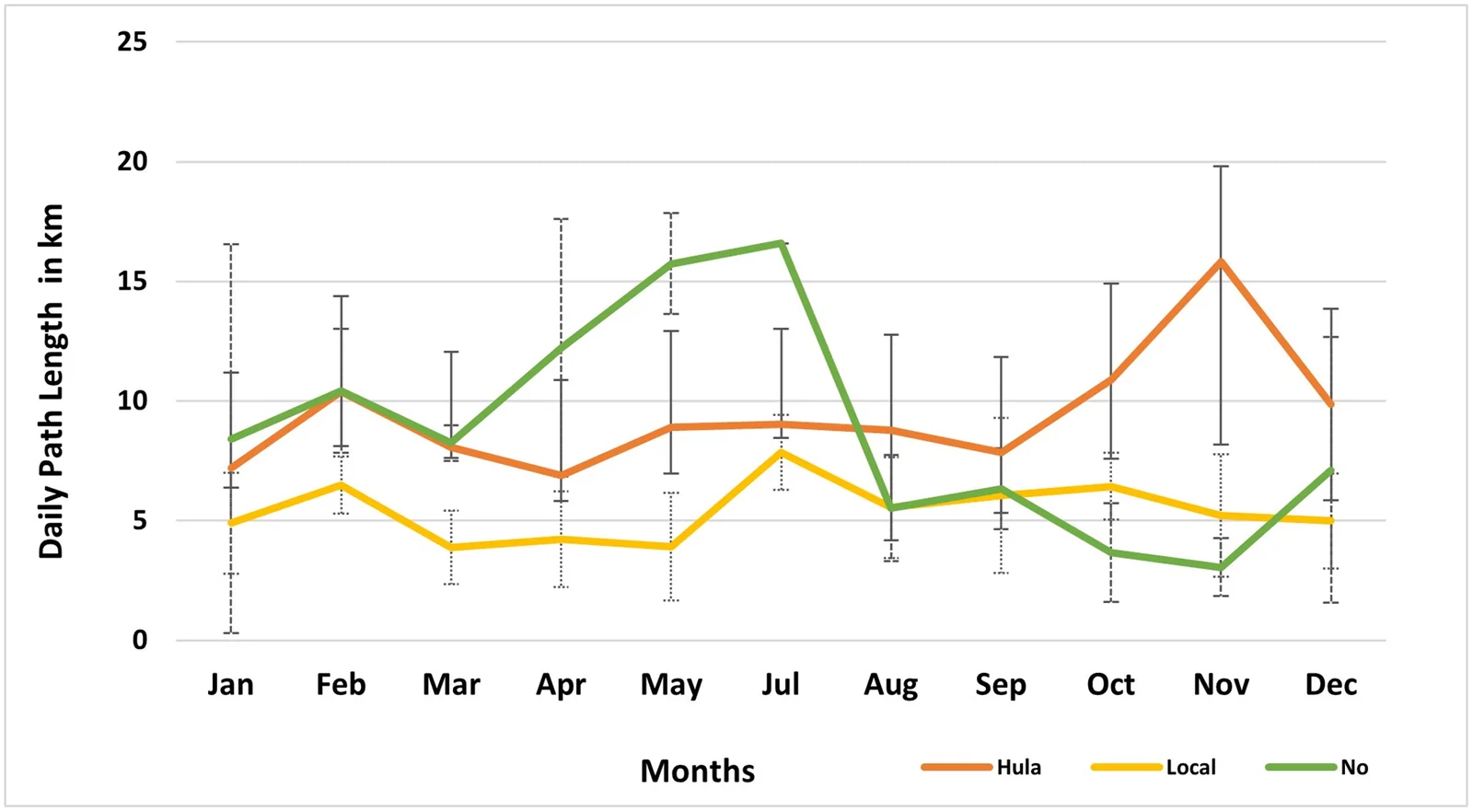
Mean path length of elephants in different drive types and months Error bar represent median with SD.

The day PL of elephants was negatively related to *hula* size (*r*_s_ = −0.095, *N* = 70, *p* < 0.05) and HS (*r*_s_ = −0.280, *N* = 70, *p* < 0.05) (Tables 1 and 2; Figure 5). While the *hula* size was positively related to the HS (*r*_*s*_ = 0.257, *N* = 70, *p* < 0.05), the duration of *hula* drive was negatively related to the *hula* size (r_s_ = −0.288, *N* = 70, *p* = 0.05). The number of elephant attacks on humans (includes both human deaths and injuries) were high (*n* = 6) on the day of the *hula* drive, followed by days 1 (*n* = 3) and 2 (*n* = 3) (Figure S3). The average area of crop land depredated immediately after the *hula* drive was relatively less, 13.39 ± 6.27_SD_ ha, which gradually increased to 28.71 ± 17.25_SD_ ha by day 4 or 5 (Figure S4).

**Table 1:** Model selection for generalized linear models explaining variation in the response variable

Model	Intercept	df	logLik	AICc	Δ	Weight
HS	2.419	3	−197.11	400.59	0	0.44
HS + DH	2.545	4	−196.78	402.18	1.59	0.20
HS + HUS	2.443	4	−197.09	402.80	2.21	0.15
NULL	2.244	2	−200.01	404.20	3.61	0.07
HS + DH + HUS	2.622	5	−196.67	404.28	3.69	0.07

(HS, herd size; DH, drive hours; HUS, *hula* size).

**Table 2:** Model-averaged effects of explanatory variables derived from generalized linear models with substantial support (ΔAICc < 2)

	Β	SE	Z	P
Intercept	2.458	0.140	17.206	<0.001
HS	−0.006	0.003	2.164	0.03
DH	−0.011	0.016	0.711	0.47

(β, beta coefficient; SE, standard error; Z, Z score; p = *p* value).

**Figure 5:**
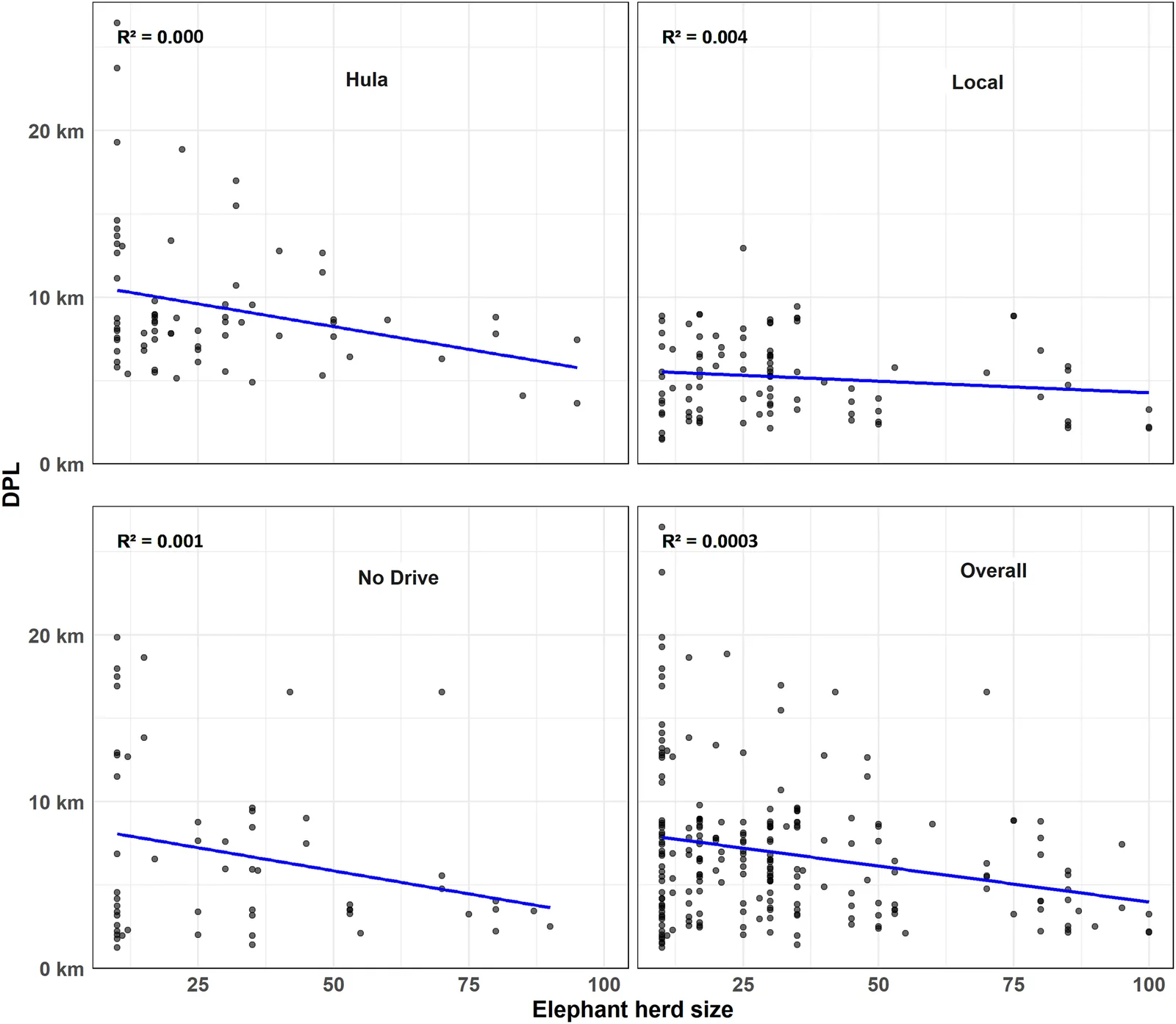
The relationship between elephant path length and their herd size in different drive types

### Assessing community response toward hula driving

Local people actively assisted in *hula* drive operations on 44.29% of incidents (*n* = 31). On the contrary, interference by local people occurred in 55.71% of operations (*n* = 39). The forms of interference included: chasing elephants in unintended directions (*n* = 16, 22.86%), taking over the operation from official handlers (*n* = 9, 12.86%), completely halting the drive (*n* = 6, 8.57%), confiscating *hula* equipment from operators (*n* = 4, 5.71%), providing assistance while simultaneously taking equipment (*n* = 3, 4.29%), and expressing frustration over delayed departmental response (*n* = 1, 1.43%; Figure S5).

## Discussion

The main aim of this study was to evaluate the effectiveness of organized hula drives as a potential conflict mitigation tool through changes in the movement behavior of the elephants, habitat use, and socio-political factors in human-dominated areas. Though these findings suggest that hula drives provide temporary relief to locals by displacing conflict from one area to another, however, it does not resolve the underlying causes of conflict. Subsequently, this study contributes to the fact that non-lethal displacement strategies should be assessed not only by their ability to immediately remove the elephants from the conflict sites but also by their long-term management and ecological significances.

The movement of elephants in South West Bengal was affected by active driving interventions, including both *hula* and local drives. Most habitats utilized by elephants comprised mosaics of forests and agriculture fields in marginal areas,^29^^,^^30^ which is consistent with use of floodplain, Sal Forest, and grassland mosaics in Bardiya National Park, Babai Valley in southwest Nepal;^31^^,^^32^ and forest-agriculture interfaces in the Simao region of Yunnan Province in China;^33^ and deciduous forest landscapes with agriculture margin in Karnataka, India.^34^^,^^35^ The forests in South Bengal are dominated by Sal trees, though it provides low-quality forage^30^ but are effectively used for taking cover. The restricted ranging, reliance on agricultural food sources and repeated negative interactions create a cycle of resource dependence and conflict.^36^^,^^37^^,^^38^ This prompts local people either to drive away the elephants from their agricultural fields.

Local drives are often observed more than *hula* drives, and are carried out only for a short distance, often from one village to nearby village. It results in continued grievances among locals as elephants continue to stay in the same region, ultimately prompting to *hula* drive by the department. Similar patterns of short-distance displacement followed by repeated crop depredation have been well documented in dry zone agricultural-forest mosaics of Sri Lanka,^15^ Amboseli, and Laikipia areas in Kenya,^39^ Leuser ecosystem of Aceh Province in Sumatra,^37^^,^^40^ parts of southern Africa,^25^^,^^41^ and Bardiya National Park and surrounding agricultural landscapes of Nepal.^31^ Recurrent community chasing offers short-term relief, but it very rarely modifies broader landscape use, leading to ongoing or spatially redistributed conflict.

*Hula* team size was administratively determined by the Forest Department and was positively associated with elephant HS, but showed no relationship with the driving distance. However, HS was not the sole determinant of operational decisions, perceived sensitivity and urgency of conflict situations shaped by prevailing socio-political pressures also played a role. Comparable administrative practices have been documented in Sri Lanka, where extensive elephant drives were initiated in response to increased public pressure despite having a limited spatial support.^18^ Also, an amount exceeding $1 million was reported for driving 250–300 elephants from an area in Sri Lanka^15^ with no long-term relief to the locals. Expenditure on *hula* drive was dependent on the *hula* size, since financial expenditure goes up directly with manpower and logistics, larger operations often cost much more without evident ecological effectiveness.^42^ These parallels indicate that organized elephant drives, irrespective of their geographical or cultural setting, tend to be expensive, reactive, and ultimately ineffective as mitigation strategies in the long term.

The day PL of elephants was much higher during the *hula* drive than in the local drive or their natural movement. Similar displacement implications have been reported in Sri Lanka, where organized drives caused elephants to travel great distances quickly and often return to their original range.^43^ Elephants in Namibia and Botswana that were exposed to severe disorientation or transboundary interference exhibited altered movement patterns and expanded territories.^41^^,^^44^ Increased daily movement distances and range shifts have been triggered by disruptions from conflict mitigation operations in Kenya’s Laikipia landscape.^45^ These enforced movements often increase stress hormone levels and energy expenditure, which can affect social stability, reproductive success, and body condition.^46^^,^^47^^,^^48^

The elevated incidence of human attacks on the day of driving, followed by a declining trend over subsequent days, highlights the immediate risk associated with *hula* operations. In India, capture and driving operations have been associated with increased aggression and a higher risk of injury to both elephants and people.^49^ Similar trends are described from southern Africa and Sri Lanka, where chase and defensive intimidation momentarily elevate the risk of conflict before elephants disperse.^18^^,^^25^ However, the observed reduction in crop depredation immediately following the drive reaching a minimum the day after suggests that *hula* is temporarily effective in suppressing crop raiding, a trend also consistent with findings from Tanzania, Zimbabwe, and Indian states of Assam and northern West Bengal that displacement-based deterrents provide short-lived relief.^39^^,^^41^^,^^50^^,^^51^ Instead of being a structural solution to conflict, these results support the idea that *hula* is a temporary, short-term mitigation technique.

The mixed response of the community to *hula* operations suggests both their requirement for the drive and their frustration of losing their crops to elephants. Gaining the local people’s confidence is essential in areas with high crop depredation by elephants.^14^^,^^15^^,^^52^ While Namibia’s community conservation areas show how participatory leadership increases tolerance regardless of ongoing damage, coordinated guarding and locally accepted preventative measures reduced aggressive behavior and improved compliance in Kenya’s Transmara region.^25^^,^^41^ Otherwise, delayed compensation has declined acceptability and led to retaliatory killings in South Asia and Africa,^53^^,^^54^^,^^55^ demonstrating that perceived injustice often led to negative attitudes among people toward elephants and conservation authorities.^52^^,^^54^

While *hula* is effective at displacing elephants over long distances, it comes with its own severe consequences for the animals, including disruption of their natural movement patterns. But the practice has its own substantial issues. *Hula* drives are not the recommended optimal mitigation approach here. Rather, our study documents their continued use, because despite their limitations, they are the only technique that has shown any measurable success in the study area, with other deterrents and physical barriers proving to be largely ineffective. Our study highlights the complex relationship between elephant behavior, strategies to reduce conflict, and socio-political factors in human-dominated areas. *Hula* is a highly reactive, socially mediated, and energy-intensive intervention that reduces conflict temporarily but does not significantly change the dynamics of long-term conflicts. Thus, empirical evaluation of *hula* drive is essential for transitioning global HEC management from reactive response to evidence-based adaptive governance.

### Limitations of the study

One limitation of this study was the lack of post-drive behavioral data for the local drive and no-drive groups. Comparisons of daily PLs between the three movement types were therefore possible during drives, but not between driven and non-driven elephants after drives.

## Resource availability

### Lead contact

Further information, requests, and inquiries should be directed to and will be fulfilled by the lead contact, Honnavalli Nagaraj Kumara (honnavallik@gmail.com).

### Materials availability

The study did not generate new materials.

### Data and code availability

• This paper does not report original code.
• The original data will be made available by the lead contact upon request.
• Any additional information required to reanalyze the data reported in this paper is available from the lead contact upon request.

## Acknowledgments

We are grateful to the office of the Chief Project director, WBFBC Project, for granting the project (project no. WBFBCP/NIT- 8(e)/2015-16). We thank Mr. S. Barari, the Principal Chief Conservator of Forests and HoFF, Mr. N.K. Pandey, the former Principal Chief Conservator of Forests and HoFF, West Bengal for granting the permission to carry out the study. We thank Dr. Pradeep Vyas, Mr. Ravi Kant Sinha, Mr. R.P. Saini, Mr. S. Sundriyal, Mr. Debal Ray, Dr. K.L. Ghosh, and Mrs. Sumana Bhattacharyya for their help and support throughout the study. We thank Dr. S. Faizi, Biodiversity Consultant-WBFBC Project, for his constant guidance. We are indebted to the Chief Conservator of Forests of all four circles, the Divisional Forest Officers of all the divisions, the Assistant Divisional Forest Officers, and the Range Officers and all the forest personnel. We thank Director, SACON, for their encouragement. We thank Santanu Mahato for his help during the study. We appreciate Mr. Debdas Mann and his family for their support during the fieldwork. We thank the anonymous reviewers for reviewing our manuscript.

## Author contributions

Conceptualization and design, H.N.K.; data provision, A.S.; formal analysis, A.S.; writing – original draft, A.S.; writing-review and editing, H.N.K.

## Declaration of interests

The authors declare no competing interests.

## STAR★Methods

### Key resources table

**Table:** 

REAGENT or RESOURCE	SOURCE	IDENTIFIER
GPS Receiver	Garmin eTrex 30s	https://www.garmin.com/en-CA/p/518048/
QGIS application (QGIS v. 3.42)	QGIS Development Team. (2018)^56^	https://qgis.org
IBM SPSS Statistics (Version 27	IBM Corp. (2020)^57^	https://www.ibm.com/support/pages/downloading-ibm-spss-statistics-27
Ranges 7 Software	South and Kenward^58^	https://www.anatrack.com/download_ranges.php
*R package*, ‘AICcmodavg’.	Mazerolle and Mazerolle^59^	https://cran.r-project.org/package=AICcmodavg

### Experimental model and study participant details

South West Bengal, encompassing an area of 37091.24 km^2^ (Figure 1), is situated between 85°30′–88°30′ E and 22°0′–24°0′ N, which includes 13 forest divisions, i.e., Rupnarayan, Purulia, Panchet, Medinipur, Kharagpur, Kangsabati South, Kangsabati North, Jhargram, Durgapur, Burdwan, Birbhum, Bankura North, and Bankura South. We conducted the study in three forest divisions: Kharagpur, Medinipur and Rupnarayan (adjacent divisions with shared boundaries). Geographically, the study area has hilly and undulating terrain on the west, and flat terrain in the east with a varying altitude of 200 m–670 m asl. The area is characterised by three major river systems, viz., Subarnarekha, Kangsabati, and Darakeswar. The temperature in the study area varies between 42°C and 46°C during summers and between 8°C and 13°C during winters. A moderate monsoon is prevalent in this area between June and September, with rainfall ranging between 1000 mm and 1500 mm.^60^ The vegetation includes a tropical dry deciduous forest type,^61^ which is dominated by Sal^30^ (*Shorea robusta*).

#### Ethics statement

The study was conducted in accordance with standard ethical practices of Salim Ali Center for Ornithology and Natural History, Wildlife Institute of India and the West Bengal forest department. All data collection methods were non-invasive and did not influence elephant behavior or functioning of the forest department. The community engagement was voluntary, and the identities of individual respondents have been anonymized to protect their privacy. No animals were captured, handled, or intentionally disturbed for this research.

### Method details

#### Elephant herd selection

We identified seven herds of elephants (size ranging from 10 to 30 individuals) that regularly used the landscape between August 2016 and July 2017. We identified individuals within each herd based on their distinct morphological features, like body scars, depigmentation patterns, variation in tail shape or length, ear notches, or any other specific marks on the body.^62^^,^^63^^,^^64^ These characteristics helped in the identification of the same individuals across repeated sightings, leading to accurate tracking of the herds. Among the identified herds, two herds (herd 1 with 10 individuals and herd 7 with 25 individuals) were using the study area for a detailed study of their habitat use and movement patterns.

#### Elephant movement and habitat utilisation

Given the large size of the study area (37,091.24 km^2^), a community-based monitoring framework was established to facilitate real-time data collection. This network included trained residents, many of whom were part of the elephant driving teams, along with elephant monitoring staff from the Forest Department. These observers reported the location of the elephant herd on spotting or while tracking them to the research team, facilitating their timely monitoring and data collection. After receiving the information on their location from the monitoring network, we proceeded to the site and confirmed herd identity using known individuals. The herds (herd-1 and herd-7) were then followed for 24 h to ensure inclusive monitoring of their movement. The location of the herd was recorded using a handheld GPS receiver (Garmin eTrex 30s) at approximately every 100 m of movement. The 100-m interval was selected to provide adequate spatial resolution for reconstructing movement paths while remaining practical under field conditions. Sequences of GPS locations were used to construct movement trajectories, quantify daily movement distances and estimate habitat usage using GIS based analyses. We collected the data on their movement and habitat utilisation between August 2017 and December 2018, except for the month of June.

During the tracking of elephants, we also documented instances where elephants were driven by residents and farmers, or organised teams coordinated by the forest department. In case of any driving occasion (irrespective of any herd), we accompanied the elephant driving teams to observe and record the details. We collected data on the type of driving team (local or organised team), description of the driving pattern, distance of the drive, size of the driving team, number of elephants, duration of the drive and the total distance traveled by elephants within 24 h following the drive. We interacted with forest department personnel and collected the information on the logistical and financial costs associated with each elephant drive. During the study period (October 2017 to December 2018), we accompanied a total of 70 organised elephant drives (out of which Herd 1 was part of 53 drives and Herd 7 of 19 drives) by the forest department and recorded the distance of the drive, the number of people involved in the drive, the reason, the material used and the cost involved for the elephant drive. Similarly, we witnessed a total of 97 local drives and also recorded the above required parameters. We monitored the hula driven elephants from a safer distance for five consecutive days after the day of *hula* drive and recorded their movement, geo-coordinates at 100-meter intervals along their movement path, changes in the group dynamics, crop depredation, human interactions, and behavior.

#### Assessing community response toward drives

We accompanied the driving teams and documented the behavioral responses of the residents toward the drives. We employed the structured observation checklist^65^^,^^66^ and recorded the inter-individual coordination, assistance to driving teams, emotional reactions toward elephants and the forest department, and support or resistance toward driving activities. We observed from a safe distance to ensure minimal disruption in the drive and reduced observer bias.

### Quantification and statistical analysis

#### Elephant movement and habitat utilisation

The geo-coordinates collected from the field during elephant movement tracking and organised driving operations were processed and analyzed using the QGIS application (QGIS v. 3.42).^56^ We placed 2 × 2 km grid cells over the study area map. A total of 1959 grids of 2 × 2 km were laid in Kharagpur, Medinipur and Rupnarayan Division. We overlaid the geocoordinates of every day walk of elephants on grid cells, and counted the total number of recorded locations in each grid cell. We graded the grid cells using the count of point locations to depict the intensity of grid use by the elephants.

We used Friedman’s two-way analysis of variance by ranks, a non-parametric substitute for repeated-measures ANOVA, to test the differences between the three types of movement (for herd 1 and 7 only). For each grid, the different driving types were collated, and the proportion of grid usage was calculated, standardizing usage intensity across movement types and allowing direct within-grid comparisons. These proportional values were then ranked within each grid, and rank sums were compared across the three movement categories to test the null hypothesis. All statistical analyses were conducted using IBM SPSS Statistics (Version 27).^57^

#### Path length estimation and movement pattern

The day path length on a 24-h temporal scale was compiled for the type of movement (for herd 1 and 7) as natural movement without interference of people, local drive when the herd was only driven by local people, and organised drive by the forest department. This dataset was subsequently processed using Ranges 7^58^ software to quantify the path length (PL). The calculation of path length in this software was done by computing the distances between consecutive geocoordinates, facilitating the estimation of the total distance traveled by the animal over a chosen time frame. Movement data spanning less than six consecutive hours were excluded from further analysis to ensure robustness of the results and to minimise any bias.

The data were collated, and differences in elephant mean path length among driving types were examined using a one-way analysis of variance (ANOVA). Driving type was included as a categorical fixed factor with three levels, and mean path length was treated as a continuous response variable. Every observation was characterised as a separate movement event. The statistical analysis was performed using IBM SPSS Statistics (Version 27). Appropriate graphs were prepared using R Statistical Software.^67^

#### Elephant drives

A Generalized Linear Modelling (GLM) framework, which utilises a Poisson error distribution with a log-link function, was used to identify the factors that influenced the distance elephants traveled during organised driving operations. The Poisson distribution was chosen because the response variable, Path Length (PL), represented count data for discrete movements. This variable showed a positively skewed distribution that is common in count processes. The log-link function helped to ensure that the predicted values stayed positive and were scaled appropriately for the data. In this analysis, Path Length was the dependent variable. Three predictors were considered, expecting to have an impact on the distance of elephant drive that included the number of individuals actively involved in driving operations of organised elephant drive (*hula* Size: HUS), the number of elephants in the group as Herd Size (HS), and total time spent on driving the elephants as Driving Hours (DH). These predictors were considered because they represented biological and management-controlled conditions that potentially influenced elephant displacement, whereas path length was treated as the response variable reflecting the resulting movement outcome. To find the best model, we built and ranked a set of candidate models using Akaike’s Information Criterion (AIC). This criterion assesses the balance between model fit and complexity to reduce the risk of overfitting. The model with the lowest AIC value and the highest weight was viewed as the best supported by the data.^59^ Model selection and ranking were carried out using the “AICcmodavg” package in R.^59^

Data recorded by following the organised elephant drive for five subsequent days were compiled, and the mean distance traveled was computed, along with summary statistics.
