# Supplementary material for: Chased and displaced: Impact of anthropogenic disturbance on habitat use and movement patterns of Asian elephants in South West Bengal, India
Source: iScience. 2026 Sep 21;29(10):117480. doi: 10.1016/j.isci.2026.117480 (PMC13626759; doi:10.1016/j.isci.2026.117480)
Supplement: Supplementary file 1 [file mmc1.pdf]

iScience, Volume *29*

Supplemental information

Chased and displaced: Impact of anthropogenic disturbance on habitat use and movement patterns of Asian elephants in South West Bengal, India

Aakriti Singh and Honnavalli Nagaraj Kumara

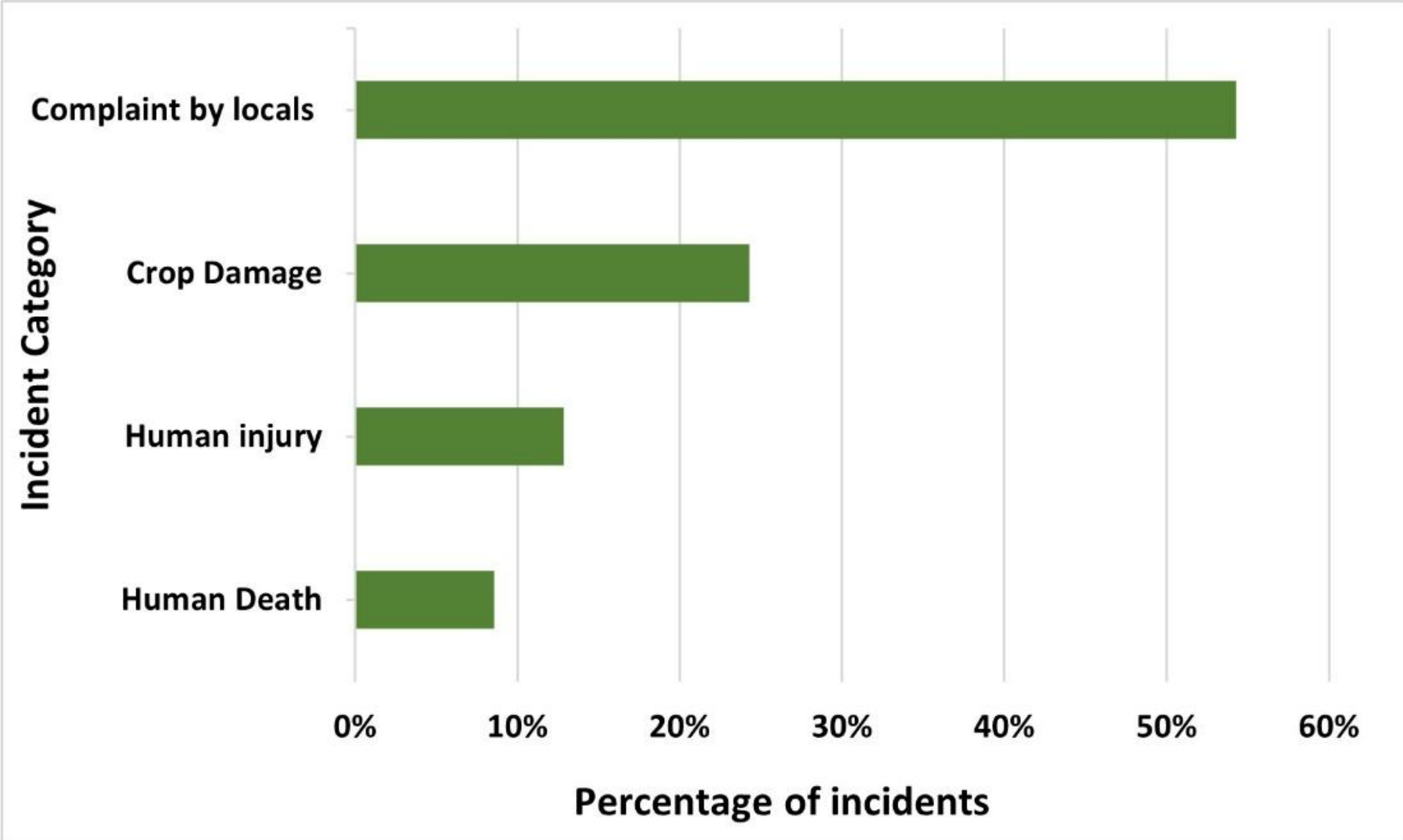

Figure S1. The reasons for driving the elephants by a hula party in South Bengal

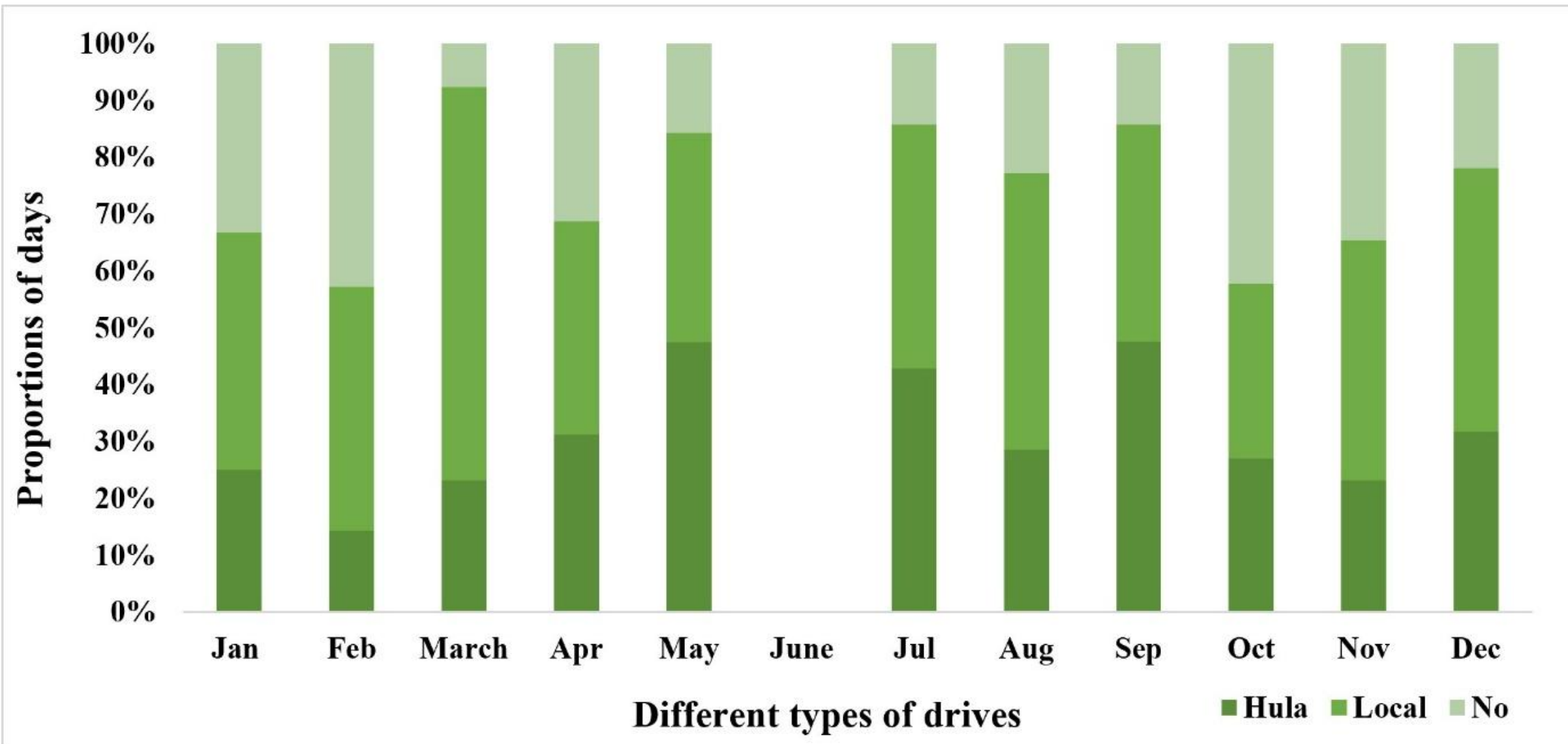

Figure S2. Month-wise movement pattern of elephants during different types of drives in South Bengal

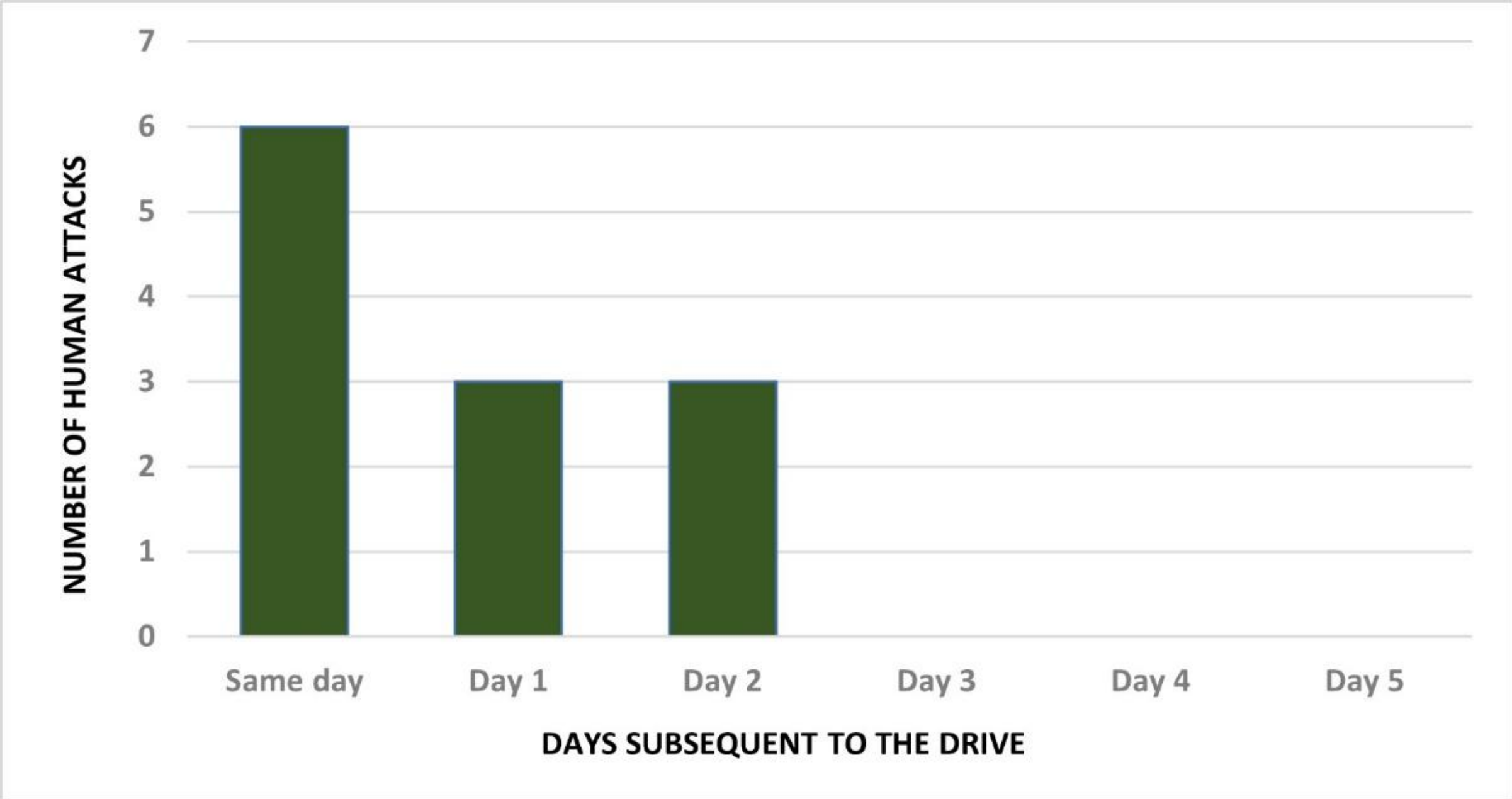

Figure S3. Graph showing the human attacks in the days (24 hr scale) after the hula drive

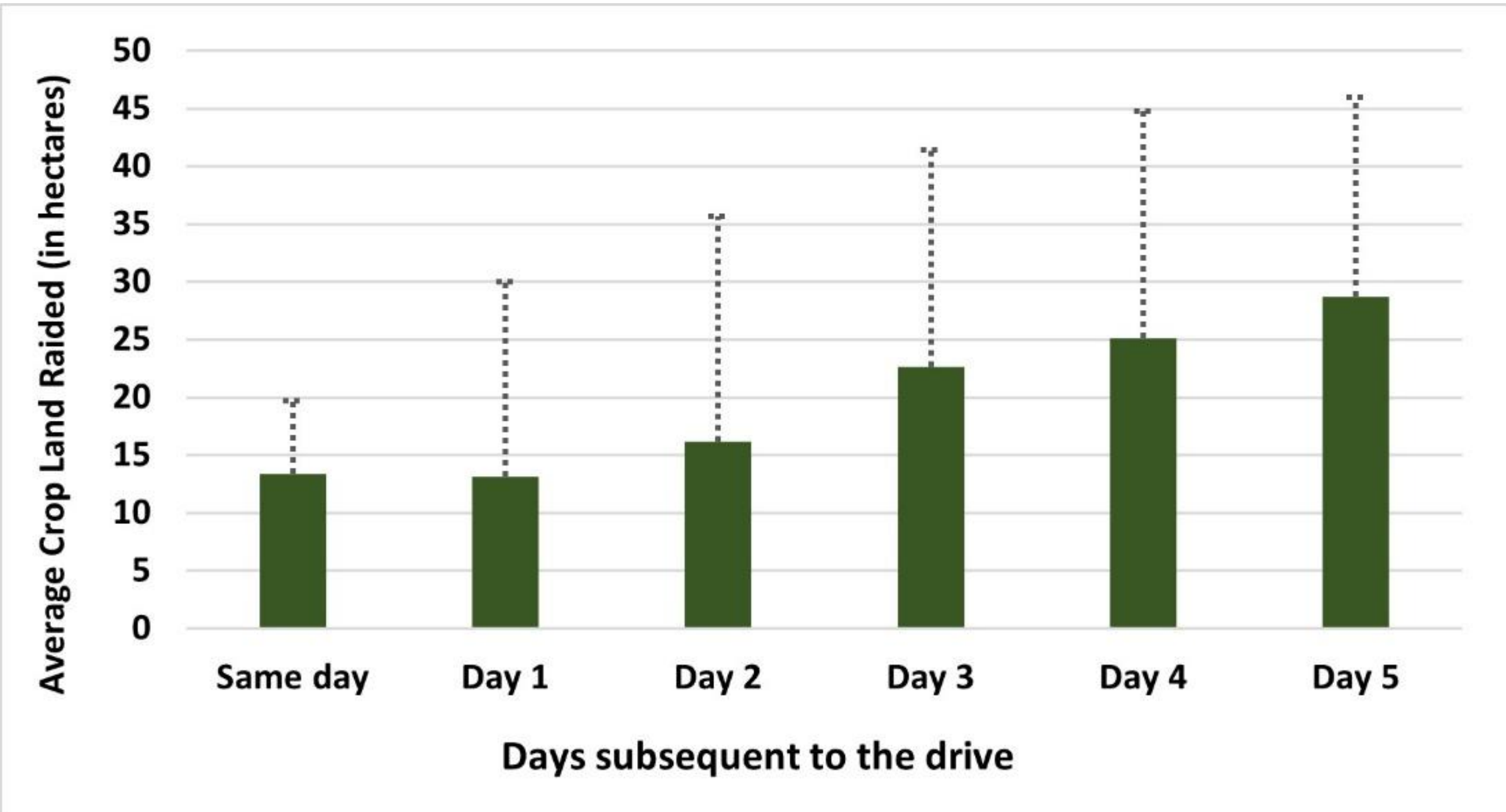

Figure S4. Graph showing the crop land depredated in the days (24 hr scale) after the hula drive

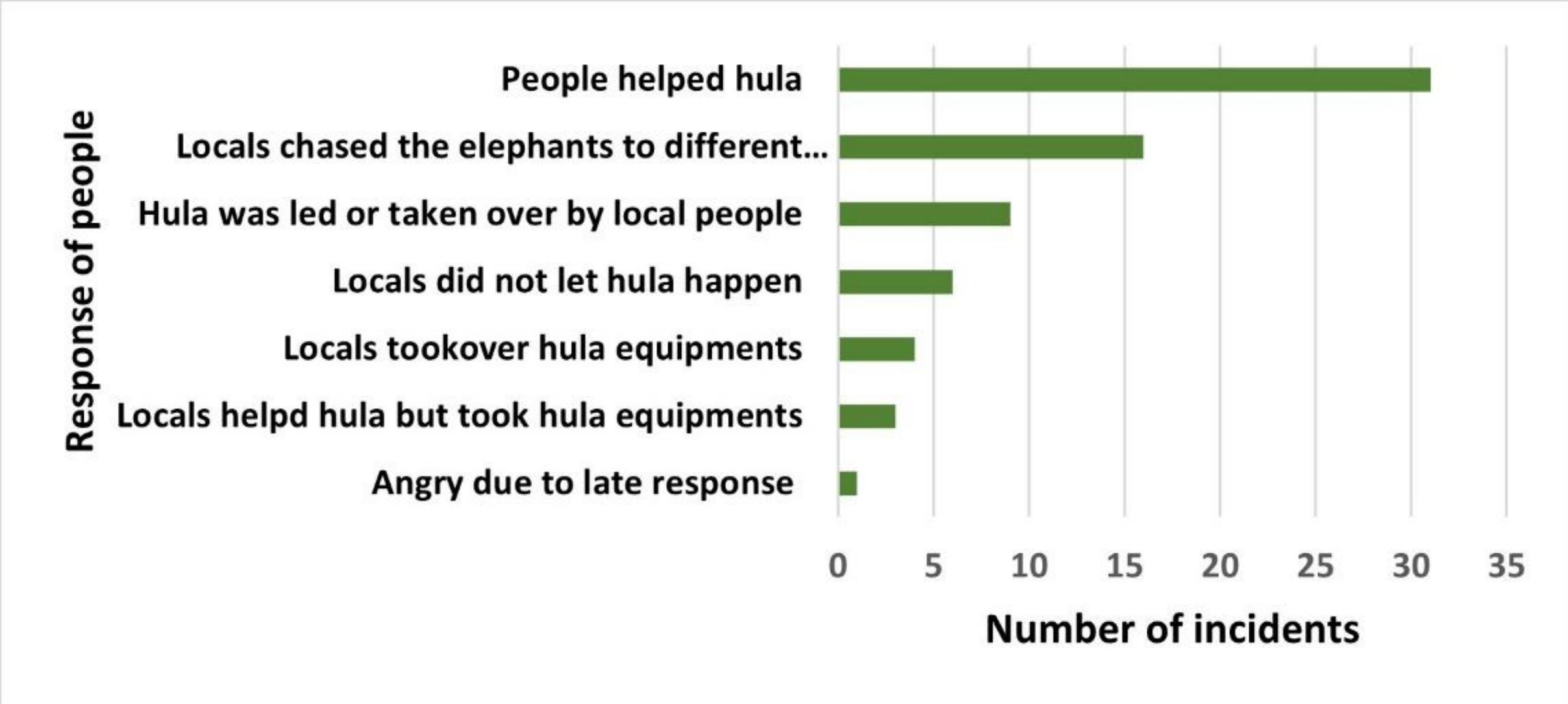

Figure S5. Responses of people towards the elephant drive in South Bengal

Supplementary Figure Legend

Supplementary Figure 1. The reasons for driving the elephants by a hula party in South Bengal

Supplementary Figure 2. Month-wise movement pattern of elephants during different types of drives in South Bengal

Supplementary Figure 3. Graph showing the human attacks in the days (24 hr scale) after the hula drive

Supplementary Figure 4. Graph showing the crop land depredated in the days (24 hr scale) after the hula drive

Supplementary Figure 5. Responses of people towards the elephant drive in South Bengal
